# De-gendering and dehumanization in mental representations of autistic men’s and women’s facial appearance

**DOI:** 10.1038/s41598-026-48196-w

**Published:** 2026-04-15

**Authors:** Matthew Elderkin, Andrew R. Todd

**Affiliations:** 1 Department of Psychology, University of California, Davis, Davis, CA 95816 USA; 2 Center for Mind and Brain, University of California, Davis, Davis, CA 95816 USA

**Keywords:** Autism, Face processing, Gender, Reverse correlation, Stereotyping, Stigma, Neuroscience, Psychology, Psychology

## Abstract

Mental representations of autistic people’s character (i.e., what they *are* like) are often negative and even dehumanizing. Yet the potential role of gender in shaping such mental representations has rarely been considered, a pattern mirroring the dearth of autism research on autistic women. Using a reverse-correlation image-classification technique, we tested whether dehumanization is evident in visualizations of autistic (vs. neurotypical) men’s and women’s facial appearance (i.e., what they *look* like) generated by non-autistic university students from the United States (*N* = 527). Results revealed that facial images of autistic men and women were mechanistically and animalistically dehumanized, but were not infantilized, more than facial images of neurotypical men and women (according to independent raters with no knowledge about whom the images depicted: *N* = 573), even for images generated by participants who explicitly disavowed such dehumanization. Visualizations of autistic men’s (women’s) faces were also ascribed fewer conventionally masculine (feminine) traits than neurotypical men’s (women’s) faces, and this de-gendering helped explain their greater dehumanization.

**Supplementary Information:**

The online version contains supplementary material available at 10.1038/s41598-026-48196-w.

## Introduction

A longstanding focus of autism research has been on developing a better understanding of the difficulties associated with autism as a clinical condition^1^. Putative difficulties aside, autistic people face challenges in the form of discrimination and social marginalization^2^. Understanding the operation of such biases toward various stigmatized groups has been a perennial aim of social psychological inquiry^3,4^. Bringing a social psychological lens to the study of autism, therefore, may hold promise for providing a richer understanding of important aspects of the autistic experience, including the stigma that autistic adults frequently endure^5^. Notably, understanding the impacts of such stigma on autistic women, long overlooked in autism research^6^, is a top priority for autism research according to autistic adults^7^.

Autistic people are commonly viewed less favorably than their neurotypical counterparts^8,9^. Depictions of autistic adults in popular media routinely highlight negative (and oftentimes unfounded) group stereotypes marked by social ineptitude and an incapacity for independence^10,11^. This negativity has important social ramifications: Non-autistic participants report less interest in interacting with—or even being around—autistic (vs. non-autistic) adults^12^. This negativity also manifests in autistic adults’ being denied full personhood, or dehumanized. Dehumanization can take several forms^13,14^. *Mechanistic* dehumanization entails denying people’s human nature and viewing them as emotionally impoverished or socially unskilled—like robots and other machines. *Animalistic* dehumanization entails denying people’s human uniqueness and viewing them as uncivilized or illogical—like lower animals. Another form of dehumanization, *infantilization*, entails denying people’s autonomy and viewing them as younger or less mature than their chronological age or life experience suggests—like children.

Existing work indicates that all three forms of dehumanization are evident in mental representations of what autistic adults *are* like (i.e., *character* representations)^15,16^. What remains unclear, and thus motivated the current investigation, is whether such dehumanization is also evident in mental representations of what autistic adults *look* like (i.e., *facial appearance* representations). With several noteworthy exceptions^17,18^, furthermore, studies documenting negativity in character representations of autistic adults either have not specified target gender or have focused on male targets exclusively. In some ways, the paucity of studies investigating mental representations of autistic women mirrors their underrepresentation in autism research more broadly^6^. Thus, another aim of this work was to examine the potential role of gender in influencing facial appearance representations of autistic adults.

Like character representations, facial appearance representations are shaped, in part, by group stereotypes^19^. So, it is perhaps unsurprising that facial appearance representations often recapitulate character representations^20–22^(but see refs^23,24^). Autism is an invisible disability characterized by its behavioral and social differences relative to the general population^25^. Despite there being no known physical markers of autism, autistic people are often told, “You don’t look autistic”^26^, which implies that mental representations of autism might include particular (facial) appearance cues. To our knowledge, however, no studies have examined what (non-autistic) perceivers think autistic men and women look like, or whether the dehumanization evident in character representations of autistic adults is also evident in facial appearance representations of autistic men and women.

One way that appearance representations of autistic adults might differ from those of neurotypical adults is in their gender typicality. Gender nonconformity in appearance and behavior is more prevalent among autistic people than in the general population^27^. Autistic people are also more likely than non-autistic people to experience gender dysphoria and to be transgender or non-binary^28,29^. Insofar as social perceivers encode these statistical regularities linking autism and gender-nonconformity^30^, they might unwittingly come to mentally represent autistic adults as looking less gender-typical, on average, than their neurotypical peers. Accordingly, a *de-gendering* hypothesis posits that faces of autistic men may be visualized as looking less conventionally masculine, and faces of autistic women may be visualized as looking less conventionally feminine, than faces of neurotypical men and women, respectively.

Gender is the social category that is most closely tied to ascriptions of humanness, and removing a person’s gender can undermine aspects of their humanity^31^. Insofar as social perceivers use gender schemas as focal lenses for making sense of other people, they may be less likely to mentally represent de-gendered targets in a way that recognizes their full humanity. De-gendered targets are also construed as socially distant and unrelatable, and these same traits are frequently ascribed to autistic adults^32,33^. So, if gendered traits are withheld from mental representations of autistic men’s and women’s appearance, as the de-gendering hypothesis posits, it might help explain any observed dehumanization in visualizations of their faces. Some evidence indicates that (non-autistic) women are animalistically dehumanized and infantilized more than (non-autistic) men^34,35^, and the dehumanization of autistic women versus men could follow a similar pattern. However, if autistic men and women are both de-gendered, gender differences in the dehumanization of autistic adults may be weaker than gender differences in the dehumanization of neurotypical adults. Notably, the de-gendering of autistic adults could have tangible implications for their dehumanization regardless of whether de-gendering reflects the accurate encoding of statistical regularities or the inaccurate construction of mental representations. We tested these lines of reasoning by investigating whether (1) facial appearance representations of autistic men and women are dehumanized more than those of neurotypical men and women, and (2) whether de-gendering contributes to such dehumanization.

## Experiment overview

In this two-phase reverse-correlation experiment, a first set of non-autistic participants (i.e., phase-1 image generators) constructed visualizations reflecting their facial appearance representation of an autistic or neurotypical man or woman and rated their assigned target group on gendered traits and measures assessing three different forms of dehumanization (mechanistic, infantilistic, and animalistic). Given claims that dehumanization reflects little more than disliking^36^, we also measured felt warmth (vs. coldness) to determine if any observed dehumanization of autistic (vs. neurotypical) men and women emerged when adjusting for general evaluations of these groups^24^. Then, different participants (i.e., phase-2 image raters), with no knowledge about whom the images depicted, rated a subset of the visualizations from each condition on the same measures from phase 1. Notably, phase-1 participants were never prompted to consider their assigned target group with respect to these measures until after generating the images. Thus, this two-phase procedure, commonly adopted in reverse-correlation research^37^, arguably affords an indirect assessment of phase-1 participants’ spontaneously generated facial appearance representations along these dimensions via phase-2 participants’ image ratings.

## Method

### Phase 1: Image Generation

**Ethics Statement.** The Institutional Review Board at the University of California, Davis approved this research and the informed consent process (#1111880-9); it was determined to involve “no more than minimal risk.” In both phase 1 and phase 2, participants read a document describing the experimental procedures and provided consent before participating. They also read a debriefing document at the end of the experimental session. All research was performed in accordance with the Declaration of Helsinki.

### Participants (Image Generators)

To our knowledge, there is no consensus about a priori power analysis calculations for phase 1 in two-phase approaches like that used here^22,38,39^. So, we used a heuristic of at least 100 participants per condition, or 400 participants in phase 1’s 2 × 2 between-subjects design. We also aimed for ≥ 80% a priori power to detect two-way interactions as small as η_p_^2^ *=* 0.03 for each measure on which the image generators directly rated their assigned target group, requiring 512 participants^40^. Anticipating exclusions, we aimed for at least 550 participants.

Due to overscheduling experimental sessions, we exceeded our target sample size. In total, 658 University of California, Davis undergraduates participated for course credit. We excluded data from participants who failed an attention check (*n* = 45), whose median response time on each trial in the image-classification task was > 4 s (*n* = 39), who responded identically to > 90% of the image-classification task trials (*n* = 3), or who, depending on condition, answered ‘1’ on an item assessing familiarity with autism (*n* = 19) or the term ‘neurotypical’ (*n* = 5). Data from participants who identified as autistic, based either on diagnosis by a medical professional or on self-diagnosis, were also excluded from analyses (*n* = 20). The final sample comprised 527 participants (*M*_age_ = 19.2, *SD*_age_ = 2.0; 75.5% women, 20.7% men, 2.5% non-binary; 47.1% Asian, Asian American, or Pacific Islander, 1.5% Black or African American, 22.4% Latina/o/e/x, 12.0% White or European American, 0.8% as Native or Indigenous American, 7.2% as another or mixed race or ethnicity; some participants did not report demographics), which afforded ≥ 80% power to detect main effects as small as η_p_^2^ = 0.025 and a two-way interaction as small as η_p_^2^ = 0.029 on the gendered traits, ascent scales, and feeling thermometer^41^. The phase-1 preregistration can be found at https://aspredicted.org/jtqy-rhbd.pdf.

### Procedure

Participants were randomly assigned to one of the 2 (Target Gender: man, woman) × 2 (Target Neurotype: autistic, neurotypical) between-subjects conditions. They completed a two-image forced-choice image-classification task^37^, generating a facial representation of a member of their assigned target group. On each of 400 trials, they selected which of two adjacent noise-laden facial images looked more like a group member. If participants did not respond within 4 s, a prompt (“Please answer the question”) appeared. Blocks of 50 trials were separated by breaks of up to 2 min each.

We created the face stimuli with the *rcicr* package (version 1.0.1)^42^ by superimposing noise pattens onto two neutral-expression base faces (one male, one female) from the Averaged Karolinska Directed Emotional Faces (AKDEF) database^43^. These gray-scaled, highly-averaged faces (the mean of all neutral-expression white male and female faces in the AKDEF) have been used frequently as base faces in reverse-correlation research^21,23,24,34^. A unique noise pattern—4,092 superimposed truncated sinusoid patches in all possible combinations of 2 cycles in 6 orientations (0°, 30°, 60°, 90°, 120°, 150°) × 5 spatial frequencies (1, 2, 4, 8, 16 patches per image) × 2 phases (0, π/2) with random contrasts—was generated for each trial and placed atop the base face to create one face stimulus. The other face stimulus in each pair was the inverse noise pattern atop the same base face, which maximizes between-face contrast.

Participants also directly reported their impressions of their assigned target group on gendered traits, mechanistic, infantilistic, and animalistic ascent scales, and a feeling thermometer. The measure of gendered traits^31,44^ entailed rating the group’s likelihood (1 = *very unlikely*, 7 = *very likely*) of possessing seven masculine traits (competitive, daring, dominant, adventurous, confident, masculine, assertive; α = 0.85) and seven feminine traits (warm, nurturing, sympathetic, gentle, feminine, supportive, affectionate; α = 0.91). Instructions were as follows:

People possess a variety of personality characteristics, or traits. Using the scale provided, please indicate the likelihood that **[autistic men/autistic women/neurotypical men/neurotypical women]** possess each trait listed below, based on your impression of the group. *There are no right or wrong answers*.

The *mechanistic* ascent scale^45^ comprised five silhouettes ranging from highly machine-like (i.e., calculator) to more human-like. The most human-like silhouette in the original mechanistic ascent scale^45^ had male-typical appearance; we used this exact silhouette in the male-target conditions. For the female-target conditions, we created a new silhouette with female-typical appearance. Instructions were as follows:

People can vary in how machine-like they seem. Some seem highly mechanical, whereas others seem no different than average humans. Using the image below, indicate on the slider how mechanical versus humanized you consider **[autistic men/autistic women/neurotypical men/neurotypical women]** to be. Ratings closer to the left side of the scale indicate more machine-like features, and ratings closer to the right side of the scale indicate more human-like features. *There is no right or wrong answer*.

**Fig. 1:**
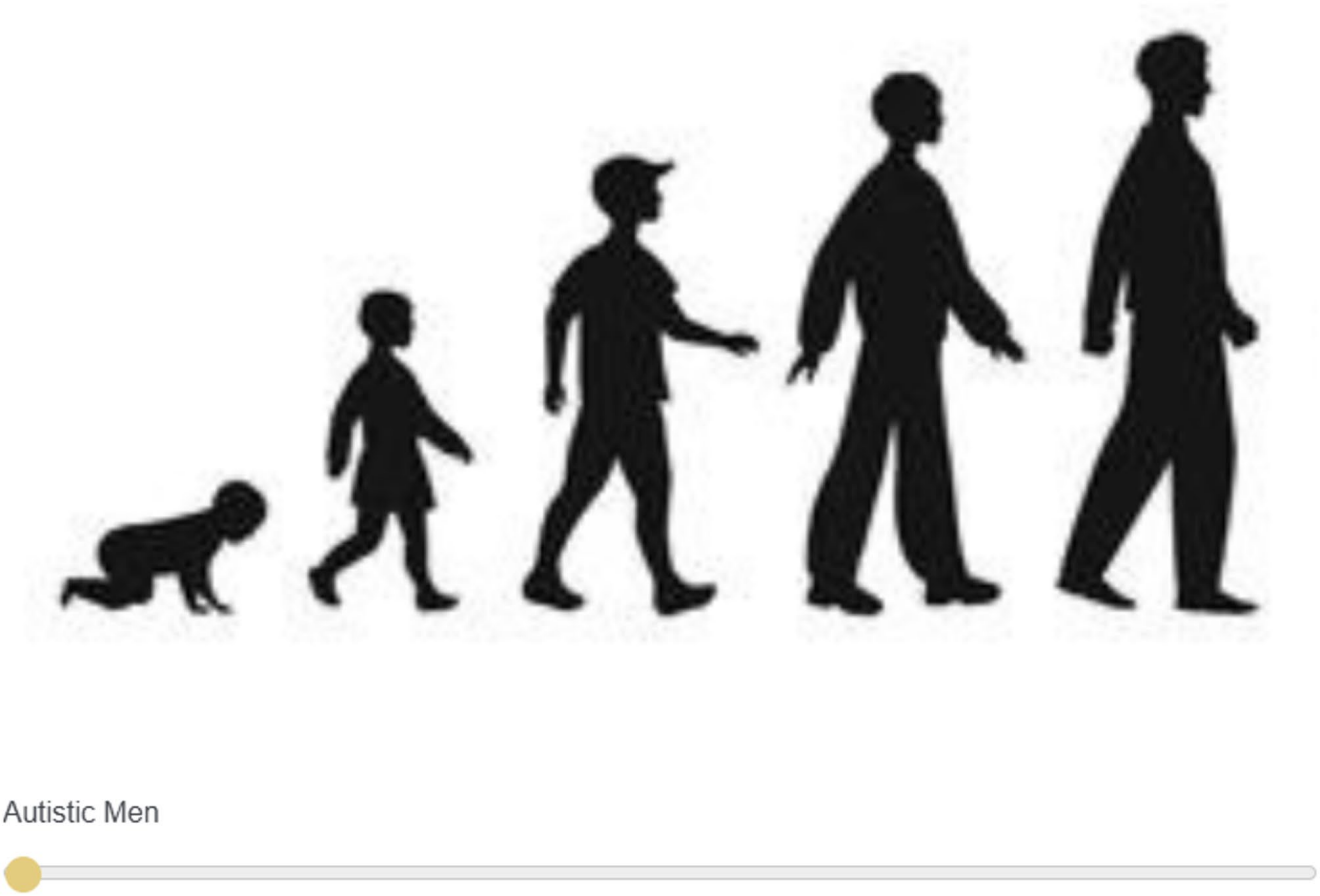
Infantilistic ascent scale (male-target version).

The *infantilistic* ascent scale comprised an analogous set of five silhouettes ranging from highly childlike (i.e., crawling baby) to more adultlike. Instructions paralleled those for the mechanistic ascent scale but with “childlike” replacing “machine-like”. The male-target version appears in Fig. 1 (see the Supplemental Materials for the female-target version).

The *animalistic* ascent scale^46^ also comprised five silhouettes, this time ranging from highly animal-like (i.e., human ancestor resembling a modern ape) to more human-like. Instructions paralleled those for the other ascent scales. Continuous slider responses for all three scales could range from 0 to 100, with lower scores (i.e., more machine-like, childlike, animal-like) indicating greater blatant dehumanization.

The feeling thermometer (“How warm or cold do you feel toward **[autistic men/autistic women/neurotypical men/neurotypical women]**?”), like the ascent scales, consisted of continuous slider responses that could range from 0 (*extremely cold*) to 100 (*extremely warm*), with lower scores indicating less positive/more negative evaluations. We included this measure^47^, commonly used to assess evaluations of autistic adults^48^, to determine if any observed dehumanization effects held when accounting for general negativity^24^.

Finally, participants rated their familiarity (1 = *extremely unfamiliar*, 7 = *extremely familiar*) with autism or the term ‘neurotypical’, depending on condition. They also completed an attention check instructing them to select “moderately disagree”, after which they reported demographics, including if they identify as autistic.

### Image processing

**Fig. 2:**
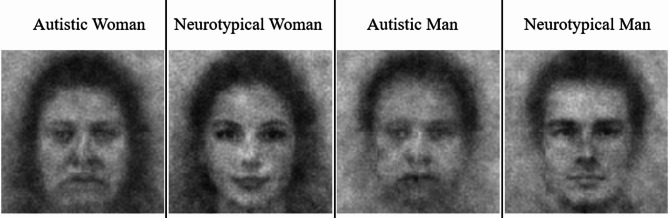
Condition-level classification images by target gender and neurotype (created with *rcicr* package^42^ and displayed for illustrative purposes only). All analyses were conducted on the participant-level images.

Using the *rcicr* package^42^, we created classification images (hereafter, ‘images’) by averaging and superimposing each participant’s selected noise patterns onto the base-face image. This procedure produced 527 participant-level images, one each from the 527 phase-1 participants whose data were retained. Condition-level images, created by averaging the images generated by all participants in each condition, appear in Fig. 2; however, to minimize Type-I error inflation from analyzing condition-level images^49^, we conducted all phase-2 analyses on participant-level images.

## Phase 2: Image Assessment

### Participants (Image Raters)

We set a target sample size of ~ 125 participants for each of four rounds of profile ratings. Anticipating exclusions, we aimed to collect data from at least 150 participants in each round.

In total, 688 different University of California, Davis undergraduates participated for course credit. We excluded data from participants who failed an attention check (*n* = 108) or who responded identically to > 90% of the images (*n* = 7). The final sample comprised 573 participants (*M*_age_ = 19.5, *SD*_age_ = 2.0; 69.3% women, 29.0% men, 1.6% non-binary; 50.1% Asian, Asian American, or Pacific Islander, 2.3% Black or African American, 19.7% Latina/o/e/x, 13.8% White or European American, 0.3% Native or Indigenous American, 9.8% another or mixed race or ethnicity; some participants did not report demographics). The smallest sample in a single round was 131 participants, which afforded ≥ 80% power to detect main effects as small as *b* = 0.13, two-way interactions as small as *b* = 0.21, and a three-way interaction as small as *b* = 0.26 on the gendered traits, and main effects as small as *b* = 1.65 and two-way interactions as small as *b* = 2.66 on the ascent scales and feeling thermometer in linear mixed-effects models^50^. The preregistrations for the gendered traits and the mechanistic and infantilistic ascent scales can be found at https://aspredicted.org/fxq2-5qvd.pdf and https://aspredicted.org/gxw4-687d.pdf. We neglected to preregister analysis plans for the animalistic ascent scale and feeling thermometer; however, we followed the same analysis plans as for the gendered traits and the mechanistic and infantilistic ascent scales.

### Procedure

Participants viewed participant-level images individually and formed an impression of each person based only on their face. To limit participant fatigue, we conducted four rounds of ratings. Of the 527 phase-1 images, phase-2 participants rated 50 or 51 randomly-selected images (evenly distributed across conditions) on one of the same measures from phase 1: (1) the three highest-loading masculine traits (competitive, daring, dominant; α = 0.83) and the three highest-loading feminine traits (warm, nurturing, sympathetic; α = 0.91) from phase 1 (*n* = 133 participants), (2) the mechanistic and infantilistic ascent scales (*n* = 160), (3) the animalistic ascent scale (*n* = 149), or (4) the feeling thermometer (*n* = 131). Finally, they completed the same attention check from phase 1 and reported demographics.

## Results

### Image-generation phase

#### Analytic strategy

We submitted ratings on the gendered traits, the three ascent scales assessing blatant dehumanization, and the feeling thermometer assessing general evaluations to separate 2 (Target Gender) × 2 (Target Neurotype) between-subjects analyses of variance (ANOVAs). Simple effects tests accompanied all significant interactions. Table 1 displays descriptive statistics for all outcome variables in the image-generation phase (top half) and the image-assessment phase (bottom half). Table 2 displays inferential statistics from omnibus analyses of all outcome variables in the image-generation phase.

**Table 1:** Mean gendered trait impressions, blatant dehumanization, and general evaluations by target gender and neurotype in the image-generation phase (top half) and image-assessment phase (bottom half). For the gendered traits, higher ratings reflect more masculine and feminine trait impressions. For blatant dehumanization, lower ratings reflect more mechanistic, infantilistic, and animalistic dehumanization. For general evaluations, lower ratings reflect less positivity/more negativity. Values in parentheses are standard deviations.

Outcome variable	Men		Women	
	Autistic	Neurotypical	Autistic	Neurotypical
Image-Generation Phase				
Gendered Traits				
Masculine	3.73 (0.99)	5.33 (0.81)	4.02 (1.07)	4.23 (0.82)
Feminine	4.20 (1.07)	3.78 (0.93)	4.28 (1.07)	5.27 (1.02)
Blatant Dehumanization				
Mechanistic	80.12 (18.68)	77.43 (20.79)	80.98 (20.10)	86.19 (20.28)
Infantilistic	57.60 (19.66)	71.17 (17.00)	60.13 (20.76)	81.59 (15.73)
Animalistic	86.17 (16.42)	77.95 (18.28)	85.61 (20.17)	88.70 (20.17)
General Evaluations	71.58 (20.30)	59.06 (18.48)	73.82 (20.82)	75.53 (16.15)
Image-Assessment Phase				
Gendered Traits				
Masculine	3.92 (0.93)	4.22 (0.94)	3.57 (0.94)	3.67 (0.90)
Feminine	2.78 (0.86)	3.33 (0.91)	2.92 (0.90)	4.31 (0.89)
Blatant Dehumanization				
Mechanistic	60.72 (22.44)	67.65 (20.84)	58.63 (22.87)	66.72 (19.64)
Infantilistic	80.33 (13.27)	79.48 (12.09)	88.62 (11.35)	71.31 (13.21)
Animalistic	68.28 (18.81)	79.07 (15.19)	58.99 (21.05)	76.46 (16.64)
General Evaluations	33.76 (11.54)	43.26 (12.08)	34.81 (12.57)	56.51 (12.80)

#### Masculine traits

Women were rated lower than men, and autistic adults were rated lower than neurotypical adults, on masculine traits. A significant interaction indicated that the gender difference (i.e., women ascribed less masculinity than men) was weaker (though still present) for autistic adults, *t*(505) = 2.47, *p* = .014, *d* = 0.22, CI_95%_ [0.04, 0.39], versus neurotypical adults, *t*(505) = 9.46, *p* < .001, *d* = 0.84, CI_95%_ [0.66, 1.02]. De-gendering of autistic men was also evident, in that they were ascribed less masculinity than neurotypical men, *t*(505) = 13.50, *p* < .001, *d* = 1.20, CI_95%_ [1.01, 1.39]. Autistic women, by contrast, did not significantly differ from neurotypical women, *t*(505) = 1.83, *p* = .068, *d* = 0.16, CI_95%_ [−0.01, 0.34].

#### Feminine traits

Women were rated higher than men, and autistic adults were rated lower than neurotypical adults, on feminine traits. A significant interaction indicated that the gender difference (i.e., women ascribed more femininity than men) emerged for neurotypical adults, *t*(501) = 11.64, *p* < .001, *d* = 1.04, CI_95%_ [0.85, 1.23], but not autistic adults, *t*(501) = 0.67, *p* = .503, *d* = 0.06, CI_95%_ [−0.12, 0.24]. Also, de-gendering of autistic women was evident, in that they were ascribed less femininity than neurotypical women, *t*(501) = 7.79, *p* < .001, *d* = 0.70, CI_95%_ [0.51, 0.88]. Autistic men, by contrast, were ascribed more femininity than neurotypical men, *t*(501) = 3.20, *p* = .002, *d* = 0.29, CI_95%_ [0.11, 0.46].

**Table 2:** Omnibus analyses of variance on gendered traits, blatant dehumanization, and general evaluations by target gender, target neurotype, and their interaction in the image-generation phase. Masculine Traits: df_2_ *=* 505. Feminine Traits: df_2_ *=* 501. Mechanistic Dehumanization: df_2_ *=* 516. Infantilization: df_2_ *=* 517. Animalistic Dehumanization: df_2_ *=* 517. General Evaluations: df_2_ *=* 518. CI_90%_ = 90% confidence interval.

Outcome Variable/Effect	*F*(1, df_2_)	*p*	η_*p*_^2^ [CI_90%_]
Masculine Traits			
Target Gender	24.18	< .001	.05 [.02, .08]
Target Neurotype	120.18	< .001	.19 [.14, .24]
Target Gender × Target Neurotype	70.72	< .001	.12 [.08, .17]
Feminine Traits			
Target Gender	74.99	< .001	.13 [.09, .18]
Target Neurotype	9.57	< .001	.02 [.004, .04]
Target Gender × Target Neurotype	59.38	< .001	.12 [.07, .15]
Mechanistic Dehumanization			
Target Gender	7.50	.006	.01 [.003, .05]
Target Neurotype	0.51	.474	< .001
Target Gender × Target Neurotype	5.06	.025	.01 [.001, .03]
Infantilization			
Target Gender	16.12	< .001	.03 [.01, .06]
Target Neurotype	117.98	< .001	.19 [.14, .23]
Target Gender × Target Neurotype	5.99	.015	.01 [.001, .03]
Animalistic Dehumanization			
Target Gender	9.69	.002	.02 [.004, .04]
Target Neurotype	2.45	.118	.01
Target Gender × Target Neurotype	11.93	< .001	.02 [.01, .05]
General Evaluations			
Target Gender	31.58	< .001	.06 [.03, .09]
Target Neurotype	10.56	.001	.02 [.004, .04]
Target Gender × Target Neurotype	18.26	< .001	.03 [.01, .06]

#### Mechanistic dehumanization

Women were mechanistically dehumanized less than men. The Target Neurotype main effect, by contrast, was not significant. A significant interaction indicated that the gender difference (i.e., women mechanistically dehumanized less than men) emerged for neurotypical adults, *t*(516) = 3.55, *p* < .001, *d* = 0.31, CI_95%_ [0.14, 0.49], but not autistic adults, *t*(516) = 0.34, *p* = .732, *d* = 0.03, CI_95%_ [−0.14, 0.20]. Also, whereas autistic men were mechanistically dehumanized no more or less than neurotypical men, *t*(516) = 1.07, *p* = .287, *d* = 0.09, CI_95%_ [−0.08, 0.27], autistic women were mechanistically dehumanized more than neurotypical women, *t*(516) = 2.14, *p* = .032, *d* = 0.19, CI_95%_ [0.01, 0.36].

#### Infantilization

Women were infantilized less than men, and autistic adults were infantilized more than neurotypical adults. A significant interaction indicated that the gender difference (i.e., women infantilized less than men) emerged for neurotypical adults, *t*(517) = 4.58, *p* < .001, *d* = 0.40, CI_95%_ [0.23, 0.58], but not autistic adults, *t*(517) = 1.11, *p* = .269, *d* = 0.10, CI_95%_ [−0.08, 0.27]. Also, the target neurotype difference (i.e., autistic adults infantilized more than neurotypical adults) emerged for both men, *t*(517) = 5.85, *p* < .001, *d* = 0.51, CI_95%_ [0.34, 0.69], and women, *t*(517) = 9.59, *p* < .001, *d* = 0.84, CI_95%_ [0.66, 1.02], but it was larger for women.

#### Animalistic dehumanization

Women were animalistically dehumanized less than men. The Target Neurotype main effect, by contrast, was not significant. A significant interaction indicated that the target gender difference (i.e., women animalistically dehumanized less than men) emerged for neurotypical adults, *t*(517) = 4.68, *p* < .001, *d* = 0.41, CI_95%_ [0.24, 0.59], but not autistic adults, *t*(517) = 0.24, *p* = .810, *d* = 0.02, CI_95%_ [−0.15, 0.19]. Also, autistic men were animalistically dehumanized *less* than neurotypical men, *t*(517) = 3.50, *p* = .001, *d* = 0.31, CI_95%_ [0.13, 0.48], whereas autistic women and neurotypical women did not significantly differ, *t*(517) = 1.36, *p* = .176, *d* = 0.12, CI_95%_ [−0.05, 0.29].

#### General evaluations

Women were rated more favorably than men, and autistic adults were rated more favorably than neurotypical adults. A significant interaction indicated that the gender difference (i.e., women rated more favorably than men) emerged for neurotypical adults, *t*(518) = 7.04, *p* < .001, *d* = 0.62, CI_95%_ [0.44, 0.79], but not autistic adults, *t*(518) = 0.95, *p* = .345, *d* = 0.08, CI_95%_ [−0.09, 0.26]. Additionally, the neurotype difference (i.e., autistic adults rated more favorably than neurotypical adults) emerged for men, *t*(518) = 5.24, *p* < .001, *d* = 0.46, CI_95%_ [0.29, 0.63], but not women, *t*(518) = 0.74, *p* = .462, *d* = 0.06, CI_95%_ [−0.11, 0.24].

#### Blatant dehumanization adjusting for general evaluations

We next tested whether the above findings held after adjusting for general evaluations, summarizing the results here and reporting details in the Supplemental Materials. When adjusting for general evaluations, the Target Gender main effects on all three forms of dehumanization were no longer significant, whereas the Target Neurotype main effect on infantilization held. Finally, the Target Gender × Target Neurotype interaction on mechanistic dehumanization and infantilization dropped to non-significance, whereas this interaction remained significant for animalistic dehumanization, as did the same underlying pattern.

#### Mediation

Finally, we conducted mediation analyses in *lavaan*^51^ to test models wherein the attenuated ascription of gender-consistent traits (masculine traits for men, feminine traits for women) mediated the effect of target neurotype (−0.5 = neurotypical, + 0.5 = autistic) on the three forms of dehumanization. We used a component approach, which requires the joint significance of both paths of an indirect effect—from predictor to mediator (*a* path) and from mediator to outcome (*b* path)—to conclude the presence of mediation^52^. We also report the indirect effect (*ab* path). Table 3 displays the path coefficients of the mediation models for each outcome in the image-generation phase (top half) and the image-assessment phase (bottom half).

**Table 3:** Parameter estimates and 95% confidence intervals for each mediation model in the image-generation phase (top half) and the image-assessment phase (bottom half). *c* = total effect. *c’* = direct effect.

Parameter	Mechanistic dehumanization	Blatant infantilization	Animalistic dehumanization
Image-Generation Phase			
*a* *b* *c* *c’* *ab*	−1.27 [−1.45, −1.10] 4.34 [2.62. 6.06] −1.74 [−5.24, 1.77] 3.79 [−0.27, 7.85] −5.52 [−7.84, −3.21]	−1.28 [−1.45, −1.10] 4.35 [2.75, 5.95] −17.52 [−20.79, −14.25] −11.97 [−15.76, −8.19] −5.55 [−7.73, −3.73]	−1.28 [−1.45, −1.11] 2.07 [0.41, 3.73] 1.99 [−1.33, 5.31] 4.65 [0.72, 8.57] −2.65 [−4.81, −0.50]
Image-Assessment Phase			
*a* *b* *c* *c’* *ab*	−0.96 [−1.09, −0.84] 2.02 [0.97, 3.07] −7.26 [−8.88, −5.66] −5.32 [−7.19, −3.44] −1.95 [−2.99, −0.90]	−0.96 [−1.09, −0.84] −4.10 [−5.01, −3.18] 9.48 [8.01, 10.94] 5.53 [3.90, 7.16] 3.94 [2.92, 4.97]	−0.96 [−1.09, −0.84] 3.53 [2.46, 4.61] −13.15 [−14.82, −11.47] −9.74 [−7.82, −11.66] −3.41 [−4.54, −2.27]

#### Mechanistic dehumanization

Despite the absence of a significant total effect of target neurotype on the mechanistic ascent scale (*c* path), we tested whether target neurotype had an indirect effect on mechanistic dehumanization through gender-consistent trait ascriptions^53^. Target neurotype predicted the ascription of fewer gender-consistent traits (*a* path), and possessing fewer gender-consistent traits predicted greater mechanistic dehumanization (i.e., lower scores on the mechanistic ascent scale; *b* path), thereby satisfying both criteria of the component approach to mediation^52^. The indirect effect through gender-consistent traits (*ab* path) was also significant. Thus, the data support a model wherein fewer gender-consistent traits were ascribed to autistic (vs. neurotypical) adults, and these weakened gender-consistent trait ascriptions, in turn, predicted greater mechanistic dehumanization.

#### Infantilization

Target neurotype predicted the ascription of fewer gender-consistent traits (*a* path), and possessing fewer gender-consistent traits predicted greater infantilization (*b* path). The indirect effect through gender-consistent traits was also significant (*ab* path). Thus, the data support a model wherein the attenuated ascription of gender-consistent traits helps explain the infantilization of autistic (vs. neurotypical) adults.

#### Animalistic dehumanization

Despite the absence of a significant total effect of target neurotype (*c* path), we tested whether target neurotype had an indirect effect on animalistic dehumanization through gender-consistent trait ascriptions. Target neurotype predicted the ascription of fewer gender-consistent traits (*a* path), and possessing fewer gender-consistent traits predicted greater animalistic dehumanization (*b* path), thereby satisfying both criteria for the component approach to mediation. The indirect effect through gender-consistent traits (*ab* path) was also significant. Thus, the data support a model wherein fewer gender-consistent traits were ascribed to autistic (vs. neurotypical) adults, and these weakened gender-consistent trait ascriptions, in turn, predicted greater animalistic dehumanization.

### Image-assessment phase

#### Analysis strategy

Using the *lme4*^54^ and *lmerTest*^55^ packages, we fit separate linear mixed-effects models for image raters’ assessments of the images on each of masculine and feminine traits, the three forms of dehumanization, and general evaluations. All models included fixed effects for Target Gender (−0.5 = men, + 0.5 = women), Target Neurotype (−0.5 = neurotypical, + 0.5 = autistic), and their interaction. We began with the maximal random-effects structure^56^ and downsized to solve model non-convergence and singularity issues. The final random-effects structure for all models included by-image and by-rater random intercepts and by-rater random slopes. Table 4 displays inferential statistics from omnibus analyses for all outcome variables in the image-generation phase.

**Table 4:** Omnibus linear mixed-effects modeling analyses of gendered traits, blatant dehumanization, and general evaluations by target gender, target neurotype, and their interaction in the image-assessment phase. CI_95%_ = 95% confidence interval.

Outcome Variable/Effect	*b *[CI_95%_]	*t*	df	*p*
Masculine Traits				
Target Gender	−0.42 [−0.50, −0.34]	−10.14	511.64	< .001
Target Neurotype	−0.18 [−0.26, −0.10]	−4.48	512.64	< .001
Target Gender × Target Neurotype	0.17 [−0.02, 0.35]	1.78	269.55	.076
Feminine Traits				
Target Gender	0.55 [0.45, 0.66]	10.04	516.24	< .001
Target Neurotype	−0.97 [−1.07, −0.86]	−17.50	516.42	< .001
Target Gender × Target Neurotype	−0.76 [−1.01, −0.52]	−6.13	405.02	< .001
Mechanistic Dehumanization				
Target Gender	−1.63 [−3.02, −0.24]	−2.30	476.25	.022
Target Neurotype	−7.71 [−9.11, −6.32]	−10.86	477.75	< .001
Target Gender × Target Neurotype	−1.34 [−4.56, 1.88]	−0.82	293.52	.415
Infantilization				
Target Gender	−0.06 [−1.31, 1.18]	−0.10	510.82	.923
Target Neurotype	9.20 [7.95, 10.44]	14.49	511.67	< .001
Target Gender × Target Neurotype	16.35 [13.35, 19.36]	10.67	407.42	< .001
Animalistic Dehumanization				
Target Gender	−6.63 [−7.47, −4.72]	−8.70	514.38	< .001
Target Neurotype	−13.98 [−15.35, −12.62]	−20.07	514.54	< .001
Target Gender × Target Neurotype	−6.58 [−9.51, −3.65]	−4.40	406.97	< .001
General Evaluations				
Target Gender	7.21 [5.92, 8.50]	10.98	515.22	< .001
Target Neurotype	−15.55 [−16.82, −14.26]	−23.77	515.81	< .001
Target Gender × Target Neurotype	−12.03 [−14.83, −9.11]	−8.41	328.33	< .001

#### Masculine traits

Images of women were rated lower than images of men, and images of autistic adults were rated lower than images of neurotypical adults, on masculine traits. Although the interaction was not significant, we examined the underlying simple effects for evidence of de-gendering of autistic men. Indeed, images of autistic men were ascribed less masculinity than images of neurotypical men, *b* = 0.27, CI_95%_ [0.14, 0.29], *t*(441.95) = 4.23, *p* < .001. Images of autistic women, by contrast, did not significantly differ from images of neurotypical women, *b* = 0.10, CI_95%_ [−0.02, 0.22], *t*(425.99) = 1.67, *p* = .096.

#### Feminine traits

Images of women were rated higher than images of men, and images of autistic adults were rated lower than images of neurotypical adults, on feminine traits. A significant interaction indicated that the target gender difference (i.e., images of women ascribed more femininity than images of men) was weaker (though still present) for images of autistic adults, *b* = 0.17, CI_95%_ [0.01, 0.34], *t*(516.24) = 2.08, *p* = .038, than for images of neurotypical adults, *b* = 0.94, CI_95%_ [0.87, 1.10], *t*(516.24) = 11.29, *p* < .001. De-gendering of autistic women was also evident, in that images of autistic women were ascribed less femininity than images of neurotypical women, *b* = −1.35, CI_95%_ [−1.51, −1.19], *t*(516.42) = −16.48, *p* < .001. Images of autistic men were also ascribed less femininity than images of neurotypical men, *b* = −0.58, CI_95%_ [−0.42, −0.75], *t*(516.42) = −6.88, *p* < .001, though this simple effect was considerably smaller than that for images of women.

#### Mechanistic dehumanization

Images of women were mechanistically dehumanized more than images of men, and images of autistic adults were mechanistically dehumanized more than images of neurotypical adults. The two-way interaction was not significant.

#### Infantilization

The Target Gender main effect was not significant; however, images of autistic adults were infantilized *less* than images of neurotypical adults. A significant interaction indicated that images of neurotypical women were infantilized more than images of neurotypical men, *b* = −8.24, CI_95%_ [−10.17, −6.30], *t*(510.82) = −8.35, *p* < .001, whereas images of autistic women were infantilized less than images of autistic men, *b* = 8.12, CI_95%_ [6.15, 10.08], *t*(510.82) = 8.09, *p* < .001. Furthermore, whereas images of autistic men were infantilized no more or less than images of neurotypical men, *b* = 1.02, CI_95%_ [−0.96, 3.01], *t*(511.67) = 1.01, *p* = .313, images of autistic women were infantilized less than images of neurotypical women, *b* = 17.38, CI_95%_ [15.46, 19.29], *t*(511.67) = 17.75, *p* < .001.

#### Animalistic dehumanization

Images of women were animalistically dehumanized more than images of men, and images of autistic adults were animalistically dehumanized more than images of neurotypical adults. A significant interaction indicated that the gender difference (i.e., images of women animalistically dehumanized more than images of men) emerged for images of neurotypical adults, *b* = −2.81, CI_95%_ [−4.81, −0.76], *t*(514.38) = −2.74, *p* = .006, and autistic adults, *b* = −9.38, CI_95%_ [−11.39, −7.37], *t*(514.38) = −9.15, *p* < .001, but it was larger for the latter. Furthermore, the neurotype difference (i.e., images of autistic adults animalistically dehumanized more than images of neurotypical adults) emerged for images of men, *b* = −10.70, CI_95%_ [−12.73, −8.66], *t*(514.54) = −10.29, *p* < .001, and women, *b* = −17.27, CI_95%_ [−19.24, −15.30], *t*(514.54) = −17.20, *p* < .001, but it was larger for the latter.

#### General evaluations

Images of women were rated more favorably than images of men, and images of autistic adults were rated less favorably than images of neurotypical adults. A significant interaction indicated that the gender difference (i.e., images of women rated more favorably than images of men) emerged for images of neurotypical adults, *b* = 13.22, CI_95%_ [11.33, 15.12], *t*(515.22) = 13.69, *p* < .001, but not for images of autistic adults, *b* = 1.19, CI_95%_ [−0.76, 3.11], *t*(515.22) = 1.23, *p* = .221. Furthermore, the neurotype difference (i.e., images of autistic adults rated less favorably than images of neurotypical adults) emerged for images of men, *b* = −9.53, CI_95%_ [−11.46, −7.60], *t*(515.81) = −9.68, *p* < .001, and women, *b* = −21.56, CI_95%_ [−23.43, −19.69], *t*(515.81) = −22.62, *p* < .001, but it was larger the latter.

#### Blatant dehumanization adjusting for general evaluations

We next tested whether the above findings held after adjusting for general evaluations, summarizing the results here and reporting details in the Supplemental Materials. When adjusting for general evaluations, the Target Gender and Target Neurotype main effects held for all three forms of dehumanization. The Target Gender × Target Neurotype interaction on infantilization also held, as did the same underlying patterns; however, this same interaction on animalistic dehumanization dropped to non-significance.

#### Mediation

Next, we conducted mediation analyses using *lavaan*^51^ to test models wherein the attenuated ascription of gender-consistent traits (masculine traits for men, feminine traits for women) to the facial images mediated the effect of target neurotype (−0.5 = neurotypical, + 0.5 = autistic) on the dehumanization of those images. We collapsed across image raters and used the mean image rating on each outcome variable and mediator. We again used a component/joint-significance-testing approach^52^ and also report the indirect effect (*ab* path).

#### Mechanistic dehumanization

Target neurotype predicted the ascription of fewer gender-consistent traits (*a* path), and possessing fewer gender-consistent traits predicted greater mechanistic dehumanization (i.e., lower scores on the mentalistic ascent scale; *b* path), thereby satisfying both criteria of the component approach to mediation^52^. The indirect effect through gender-consistent traits was also significant (*ab* path). Thus, the data support a model wherein the attenuated ascription of gender-consistent traits helps explain the greater mechanistic dehumanization of images of autistic (vs. neurotypical) adults.

#### Infantilization

Target neurotype predicted the ascription of fewer gender-consistent traits (*a* path); however, unlike the pattern for mechanistic dehumanization, possessing fewer gender-consistent traits predicted *less* infantilization (i.e., higher scores on the infantilistic ascent scale; *b* path). The indirect effect through gender-consistent traits was also significant (*ab* path). Thus, the data support a model wherein the attenuated ascription of gender-consistent traits helps explain the *weaker* infantilization of images of autistic (vs. neurotypical) adults.

#### Animalistic dehumanization

Target neurotype predicted the ascription of fewer gender-consistent traits (*a* path), and possessing fewer gender-consistent traits predicted greater animalistic dehumanization (i.e., lower scores on the animalistic ascent scale; *b* path). The indirect effect through gender-consistent traits was also significant (*ab* path). Thus, the data support a model wherein the attenuated ascription of gender-consistent traits helps explain the animalistic dehumanization of images of autistic (vs. neurotypical) adults.

### Correspondence between image-generation phase and image-assessment phase ratings

Finally, to investigate the relationship between the group impressions directly reported by image generators in phase 1 and the ratings of their generated images in phase 2, we examined correlations between the phase-1 and phase-2 ratings. There was a medium-sized positive correlation for feminine traits, *r*(512) = 0.38, CI_95%_ [0.30, 0.45], *p* < .001, and small positive correlations for masculine traits, *r*(515) = 0.12, CI_95%_ [0.04, 0.21], *p* = .004, and general evaluations *r*(522) = 0.12, CI_95%_ [0.04, 0.21], *p* = .005. This same correlation was not significant for animalistic dehumanization, *r*(521) = − 0.02, CI_95%_ [−0.10, 0.07], *p = *.781, or mechanistic dehumanization, *r*(520) = − 0.03, CI_95%_ [−0.11, 0.06], *p* = .552; however, there was a medium-sized *negative* correlation for infantilization, *r*(521) = −0.28, CI_95%_ [−0.36, −0.20], *p* < .001.

## Discussion

Facial appearance representations of autistic men and women generated by non-autistic undergraduates in the United States were mechanistically and animalistically dehumanized more than facial appearance representations of neurotypical men and women, conceptually replicating prior work documenting dehumanization in character representations of autistic people^15,16^. Importantly, this dehumanization was evident for autistic (vs. neurotypical) men *and* women and even when accounting for general evaluations of these groups. Contrary to prior work documenting infantilization in character representations of autistic adults^16^, however, facial appearance representations of autistic adults (particularly autistic women) were infantilized *less* than those of neurotypical adults. Visualizations of autistic (vs. neurotypical) adults’ faces were also de-gendered (i.e., autistic men and women were visualized as less conventionally masculine and less conventionally feminine, respectively), and this de-gendering helped explain their greater mechanistic and animalistic dehumanization.

Notably, these dehumanized facial representations were evident even among image generators who explicitly disavowed such dehumanization. That is, although the conclusions drawn from reverse-correlation paradigms often match those drawn from self-reported impression measures^20–22^, we found strikingly different patterns in image generators’ explicit impressions of autistic (vs. neurotypical) men and women and their visualizations of these groups’ faces, as has also been documented for some other social groups (e.g., immigrants^23^, Arab people^24^). Specifically, whereas de-gendering was evident in image generators’ self-reported impressions of autistic (vs. neurotypical) adults, mechanistic dehumanization was not. The image generators also explicitly infantilized autistic men and women more, and animalistically dehumanized autistic men and women less, than their neurotypical counterparts, contrary to assessments of the facial images they generated.

The size of the correlations between the phase-1 and phase-2 ratings suggests varying correspondence between directly-assessed character representations (gleaned from the image generators’ explicit impressions) and indirectly-assessed facial appearance representations (gleaned from the image raters’ impressions of the image generators’ visualizations). The strength (and direction) of the correspondence depended on the specific characteristic in question. Because the image generators’ explicit impressions of their assigned group were assessed after the image-classification task, they might have regulated their self-reported responses, resulting in small or non-significant correlations with image raters’ impressions of the facial images. The negative correlation for infantilization is consistent with the possibility that autistic adults’ character, but not their facial appearance, is infantilized. That is, whereas variants of “childlike” (e.g., “youthful”) are arguably positive descriptors of someone’s facial appearance^57^, they are much less positive descriptors of someone’s character.

Furthermore, the target neurotype effects on animalistic dehumanization and general evaluations were larger for facial images of women than for facial images of men, and only facial images of autistic women were infantilized less than those of their neurotypical counterparts. These effects further support the notion that higher ratings on the infantilistic ascent scale (i.e., less childlike) might partially reflect stronger impressions of unattractiveness. Indeed, (un)attractiveness more strongly predicts the (de)humanization of women than men^58^.

The current findings have important practical implications for how autistic adults are treated both institutionally and interpersonally. Dehumanization facilitates the endorsement of instrumental violence^59^, and infantilization is linked to worse lifelong professional success^34^. In addition, the mechanistic dehumanization of women who endure intimate partner violence can undermine their assumed suffering and can soften sentencing recommendations for the perpetrators^60^. Although little work has examined these links for autistic men and women specifically, there is ample evidence that autistic adults are more likely to be unemployed than their neurotypical peers^61^. Like children, autistic adults are also believed to be particularly vulnerable to experiencing pain, which may reflect their infantilization^62^.

Novel insights gleaned from this research notwithstanding, several limitations warrant mentioning, each suggesting directions for future research. Reverse-correlation techniques, like other indirect measures^63^, afford an unobtrusive glimpse into mental representations beyond what image generators are willing and/or able to report directly^64^. Nevertheless, such techniques still constrain participants’ ability to generate an image reflecting the “picture in their mind” of their assigned target group^65^. Indeed, our use of white European male and female base-face images, despite being the same ones used extensively in prior work assessing facial representations of various social groups^21,23,24,38^, necessarily constrained how much the visualizations could vary in their appearance. Future work could use a base-face image that is more ambiguous on various dimensions (e.g., apparent race, gender)^39,66^.

Furthermore, aspects of appearance over which people regularly exert control (e.g., hairstyle and length, clothing, accessories) are not captured by this method, despite their likely being among the appearance-based indicators that perceivers might most readily use to distinguish autistic from neurotypical adults. Future research could take a more open-ended approach to capturing appearance representations, for example, by having participants draw a relevant group member^67^.

We also cannot conclude from this experiment whether the de-gendering and dehumanization of autistic (vs. neurotypical) adults are autism-specific or generalize to a broader marginalized social category to which autistic adults belong, such as neurodivergent or disabled adults. To investigate the specificity of these findings, future research could use a neurodivergent comparison group (e.g., men and women with ADHD).

Another limitation is that our correlation-based mediation analyses cannot provide causal evidence that de-gendering explains the dehumanization of autistic (vs. neurotypical) men and women^68,69^. It is possible, perhaps even likely, that the relationship between de-gendering and dehumanization is bidirectional, with the withholding of gendered traits serving to justify dehumanization in some cases^31^. Future experiments that manipulate de-gendering (dehumanization) and measure its impact on dehumanization (de-gendering) could be useful in establishing causal (bi)directionality^69^.

Finally, we are limited by our convenience samples of non-autistic undergraduates at a university in the Western United States. Although these samples were somewhat diverse racially/ethnically, they were homogeneous in age. Thus, our findings might not reflect broader population-level visualizations of autistic (vs. neurotypical) men’s and women’s facial appearance. Mental representations of autistic adults could differ in geographical regions where knowledge about and experiences with autism are more versus less extensive than in the current work. Sampling from a broader swath of participants will surely benefit future research.

In sum, this work was inspired by recent calls to integrate social psychological insights into autism research (and vice versa)^5^ and to prioritize research on attitudes toward autistic adults (particularly autistic women^7^). Heeding such calls, we borrowed a method originally developed in cognitive psychology^64^ and commonly used in social psychology^37^—the reverse-correlation image-classification technique—to examine whether dehumanization in character representations of autistic adults is recapitulated in facial appearance representations of autistic men and women. We found that visualizations of autistic adults were mechanistically and animalistically dehumanized, but were not infantilized, more than visualizations of neurotypical adults, even among (non-autistic) participants who explicitly disavowed such dehumanization. Furthermore, our findings suggest that de-gendering in facial representations of autistic (vs. neurotypical) men and women can help explain their greater dehumanization.

## Supplementary Information

Below is the link to the electronic supplementary material.

Supplementary Material 1

## Data Availability

Study materials, data files, analysis scripts, preregistrations, and supplemental materials are available at [https://doi.org/10.17605/OSF.IO/4GDJ2](https:/doi.org/10.17605/OSF.IO/4GDJ2) .
