# Supplementary material for: De-gendering and dehumanization in mental representations of autistic men’s and women’s facial appearance
Source: Sci Rep. 2026 Apr 15;16:17442. doi: 10.1038/s41598-026-48196-w (PMC13236976; doi:10.1038/s41598-026-48196-w)
Supplement: Supplementary file 1 — Supplementary Material 1 [file 41598_2026_48196_MOESM1_ESM.pdf]

Supplemental Materials

De-Gendering and Dehumanization in Mental Representations of Autistic Men and Women's Facial Appearance

Table of Contents

Female Version of Infantilistic Ascent Scale 3

Blatant Dehumanization Adjusting for General Evaluations (Image-Generation Phase) 4

Blatant Dehumanization Adjusting for General Evaluations (Image-Assessment Phase) 5

Female Version of Infantilistic Ascent Scale

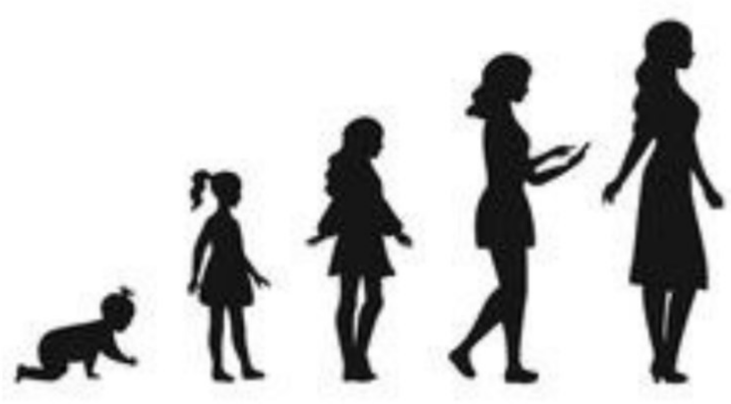

Blatant Dehumanization Adjusting for General Evaluations (Image-Generation Phase)

To determine if the reported effects for mechanistic, infantilistic, and animalistic dehumanization in the image-generation phase held after adjusting for general evaluations, we submitted the ascent scales to separate analyses of covariance (ANCOVAs). Each analysis included the same main effects and interactions from the ANOVAs, along with the feeling thermometer ratings as a covariate. We only report analyses corresponding to effects that were significant in the primary analyses reported in the main text.

Mechanistic. Neither the Target Gender main effect, $F(1, 514) = 0.03$, $p = .952$, $\eta_p^2 < .001$, nor the Target Gender × Target Neurotype interaction, $F(1, 514) = 0.95$, $p = .334$, $\eta_p^2 = .002$, remained significant after adjusting for general evaluations.

Infantilistic. Neither the Target Gender main effect, $F(1, 515) = 0.79$, $p = .358$, $\eta_p^2 = .002$, nor the Target Gender × Target Neurotype interaction, $F(1, 515) = 1.45$, $p = .229$, $\eta_p^2 = .003$, remained significant after adjusting for general evaluations. However, the Target Neurotype main effect did remain significant, $F(1, 515) = 56.78$, $p < .001$, $\eta_p^2 = .10$, $CI_{90\%}$ [.06, .14].

Animalistic. The Target Gender main effect was not significant after adjusting for general evaluations, $F(1, 515) = 0.19$, $p = .662$, $\eta_p^2 < .001$. However, the Target Gender × Target Neurotype interaction did remain significant, $F(1, 515) = 5.35$, $p = .021$, $\eta_p^2 = .01$, $CI_{90\%}$ [.001, .03], as did the same underlying patterns.

Blatant Dehumanization Adjusting for General Evaluations (Image-Assessment Phase)

To determine if the reported effects for mechanistic, infantilistic, and animalistic dehumanization in the image-assessment phase held after adjusting for general evaluations, we submitted the ascent scale ratings to separate linear regressions with main effects for Target Gender (-0.5 = men, +0.5 = women), Target Neurotype (-0.5 = neurotypical, +0.5 = autistic), and their interaction, along with the feeling thermometer ratings as a covariate. Because the participants who rated the images on the ascent scales were different from those who rated the images on the feeling thermometer, we collapsed across image raters and used the mean image rating for each outcome variable and covariate in each model. We only report analyses corresponding to effects that were significant in the primary analyses reported in the main text.

Mechanistic. The main effects of Target Gender, $b = 3.14$, $CI_{95\%}$ [0.93, 5.35], $t(522) = 2.79$, $p = .005$, and Target Neurotype, $b = 3.31$, $CI_{95\%}$ [0.90, 5.73], $t(522) = 2.69$, $p = .007$, remained significant after adjusting for general evaluations.

Infantilistic. The Target Neurotype main effect, $b = 2.48$, $CI_{95\%}$ [0.61, 4.36], $t(522) = 2.59$, $p = .009$, and the Target Gender × Target Neurotype interaction, $b = 12.86$, $CI_{95\%}$ [10.29, 15.44], $t(522) = 9.80$, $p < .001$, remained significant after adjusting for general evaluations, as did the same underlying patterns.

Animalistic. The main effects of Target Gender, $b = 9.71$, $CI_{95\%}$ [7.65, 11.77], $t(522) = 9.24$, $p < .001$, and Target Neurotype, $b = 4.99$, $CI_{95\%}$ [2.73, 7.24], $t(522) = 4.33$, $p < .001$, remained significant after adjusting for general evaluations. However, the Target Gender × Target Neurotype interaction did not, $b = 0.78$, $CI_{95\%}$ [-2.30, 3.87], $t(522) = 0.50$, $p = .619$.
